# HPLC Determination of Antilipoxygenase Activity of a Water Infusion of *Ligustrum vulgare *L. Leaves and Some of Its Constituents

**DOI:** 10.3390/molecules16108198

**Published:** 2011-09-28

**Authors:** Pavel Mučaji, Anna Záhradníková, Lýdia Bezáková, Mária Cupáková, Drahomíra Rauová, Milan Nagy

**Affiliations:** 1 Department of Pharmacognosy and Botany, Faculty of Pharmacy, Comenius University, Odbojárov 10, 832 32 Bratislava, Slovak Republic; Email: zahradnikova@fpharm.uniba.sk (A.Z.); nagy@fpharm.uniba.sk (M.N.); 2 Department of Cell and Molecular Biology of Drugs, Faculty of Pharmacy, Comenius University, Odbojárov 10, 832 32 Bratislava, Slovak Republic; Email: bezakova@fpharm.uniba.sk; 3 Toxicological and Antidoping Centre, Faculty of Pharmacy, Comenius University, Odbojárov 10, 832 32 Bratislava, Slovak Republic; Email: cupakova@fpharm.uniba.sk (M.C.); rauova@fpharm.uniba.sk (D.R.)

**Keywords:** *Ligustrum*, phenolics, inflammation, lipoxygenase, antioxidants, echinacoside, oleuropein

## Abstract

The aim of the study was a HPLC evaluation of the lipoxygenase activity inhibiting activity of a water infusion of *Ligustrum vulgare* L. leaves and selected isolates from it. The antiradical activity of the water infusion was determined using DPPH, ABTS and FRAP tests. Oleuropein and echinacoside concentrations in the water infusion were determined by HPLC. Water infusion, echinacoside and oleuropein were used for an antilipoxygenase activity assay using lipoxygenase isolated from rat lung cytosol fraction. Activity of 8-LOX, 12-LOX and 15-LOX were monitored through formation of 8-HETE, 12-HETE and 15-HETE, respectively. The water infusion exhibited the highest activity against all lipoxygenases, followed by oleuropein. Echinacoside was ineffective against LOXs in lower concentrations, while higher concentration showed similar inhibition on 8-LOX and 12-LOX. 15-LOX was affected more and the presence of echinacoside remarkably decreased its activity.

## 1. Introduction

Many species of genus *Ligustrum *have been traditionally used worldwide in folk medicine. In the folk medicine of Azerbaijan, the use of common privet (*Ligustrum vulgare* L.) (LV) leaves is linked to the treatment of hypertension [1], and is supported by recent studies of the hypotensive and diuretic effects of this plant. It was shown that different extracts of this plant act as dual angiotensin-converting enzyme and neutral endopeptidase inhibitors [2]. Common privet leaves are currently used as an oropharyngeal anti-inflammatory agent in the ethnomedicine of southern Italy and the plant is regarded as an antirheumatic in Cyprus. In Anatolia (Turkey) fresh plant leaves are still chewed to cure aphtae [3]. Chinese and Japanese medicine has used this plant due to its liver-protecting [4], antiviral [5] or anti-mutagenic effects [6]. Most of these diseases can be connected with the reactive oxygen species balance in all human tissues. Phenolic antioxidants are recognized as the main active principles in privet [7]. In connection to the usage of privet in traditional medicine we decided to evaluate the potential anti-inflammatory activity of this plant and some its constituents. Among the different anti-inflammatory test systems available, *in vitro* assay of inhibitory activity on lipoxygenase was chosen.

A variety of redox active compounds have been identified as inhibitors of lipoxygenases. Lipoxygenase (LOX) is the key enzyme in the biosynthesis of leukotrienes, which are postulated to play an important role in the pathophysiology of several inflammatory diseases since the products of lipoxygenase catalyzed oxygenation (as hydroperoxyeicosatetraenoic acids—HPETEs, hydroxy-eicosatetraenoic acids—HETEs, leukotrienes and lipoxins) seem to be involved in inflammatory reactions (rheumatoid arthritis and psoriasis). There is also good evidence that leukotrienes are mediators of asthmatic responses, glomerular nephritis, myocardial ischemia and cancer [8].

**Figure 1:**
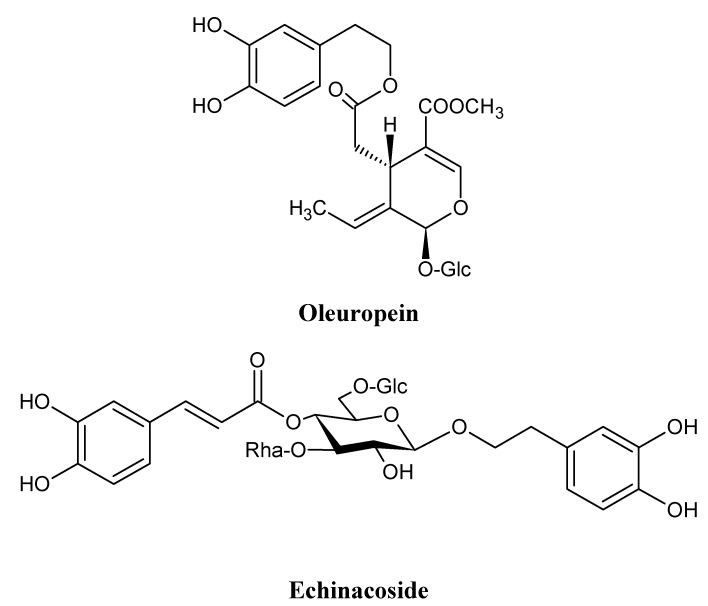
Structures of oleuropein and echinacoside.

In previous work we have reported the inhibitory activity of a water infusion of *Ligustrum vulgare* L. leaves, and that of two isolates from the infusion, echinacoside and oleuropein (Figure 1) on lipoxygenase isolated from rat lung cytosol fraction as determined by a spectrophotometric method [9].

The aim of this study was an HPLC evaluation of the activity of the previously mentioned components and water infusion against LOXs isolated from rat lung cytosol fraction and their influence on different lipoxygenases (8-LOX, 12-LOX and 15-LOX). The concentrations of oleuropein and echinacoside in the water infusion were determined by HPLC and the antiradical activity of the samples was determined using 2,2-diphenyl-1-picrylhydrazyl (DPPH) radical, 2-2′-azinobis-(3-ethylbenzo-thiazoline-6-sulfonic acid (ABTS) radical and ferric reducing ability of plasma (FRAP) tests.

## 2. Results and Discussion

Eicosanoids have potent and diverse biological activities in cell proliferation, tissue repairing, blood clotting, blood vessel permeability, and immune cell behavior. These compounds also play a very important role in the pathogenesis of inflammation and cancer. For example, prostaglandin PGE_(2)_, 5-HETE and 12-HETE have been found to stimulate the proliferation of various cancer cells and to promote tumor development. In contrast, 13-hydroxyoctadecadienoic acid and 15-HETE, appear to inhibit the growth of colon and prostate cancer cells. Increased production of leukotriene LTB_(4)_, 5-HETE, and 12-HETE has also been reported in the mucosa of colon cancer patients and the saliva of oral cancer patients. Because eicosanoids appear to have both negative and positive modulatory effects, they seem to be vital in the prevention of inflammation-related diseases, including cancer [10].

We have previously reported the inhibitory activity of a water infusion of *Ligustrum vulgare* L. leaves and its components echinacoside and oleuropein on lipoxygenase, where activity of LOX was monitored as an increase in the UV absorbance at 234 nm that reflected the formation of hydro-peroxylinoleic acid [9]. In that work all tested samples exhibited remarkable inhibitory effect on LOX, with IC_50_ values of 82.2 μmol L^−1^ for oleuropein, 296 μmol L^−1^ for echinacoside, 0.388 mg L^−1^ for water infusion of LV leaves and competitive character of inhibition of all tested inhibitors. In the present study we evaluate the influence of mentioned infusion and its constituents on some different lipoygenases by an HPLC method.

The analytical procedure described in Experimental was applied for determination of echinacoside and oleuropein in the lyophilized sample of infusion of *L. vulgare *leaves. The analyzed sample contained 72.29 μg of echinacoside and 145.45 μg of oleuropein in 5 mg of lyophilizate. Standardized sample was further used for determination of the inhibitory activity on lipoxygenase isolated from rat lung cytosol fraction and antiradical activity using DPPH, ABTS and FRAP tests because many antioxidants are known inhibitors of this enzyme (Table 1). The antioxidative activity of oleuropein and echinacoside is well known and described in literature [11,12,13,14] and therefore we have not evaluated it.

**Table 1:** SC_50_ values of antiradical activity of lyophilized infusion from *Ligustrum vulgare *leaves compared with ascorbic acid.

Method	*L. vulgare infusion* SC_50_ [µg.mL^−1^]	Ascorbic acid SC_50_ [µg.mL^−1^]
DPPH	20.68	1.96
ABTS	110.14	23.84
FRAP	1004.12	238.89

Activity of lipoxygenase was affected in a dose-dependent manner, based on used inhibitor, as shown in Figure 2 (for concentrations used and SD values see Table 2 and Table 3). The most potent inhibitor was *Ligustrum vulgare* infusion, which exhibited 76% inhibiting activity against 8-LOX and more than 60% against 12-LOX and 15-LOX, respectively. Neither of the compounds isolated from *L. vulgare *reached this level of activity. Oleuropein inhibits about 40% activity of all three lipoxygenases, while echinacoside was ineffective in lower concentrations. At higher concentration it showed a similar inhibition of 8-LOX and 12-LOX (28.7% and 27%, respectively). 15-LOX was more sensitive, and the presence of echinacoside decreased its activity to 47%. The different inhibitory effectiveness of the isolated compounds and water infusion on lipoxygenase activity could be influenced by the presence of other constituents in the prepared infusion. It is known that plant polyphenols exhibit inhibitory effects on prooxidative enzymes such as xanthine oxidase, myeloperoxidase or lipoxygenases and this fact may additionally contribute to the potential inhibitory effect of the water infusion observed in our work.

**Figure 2:**
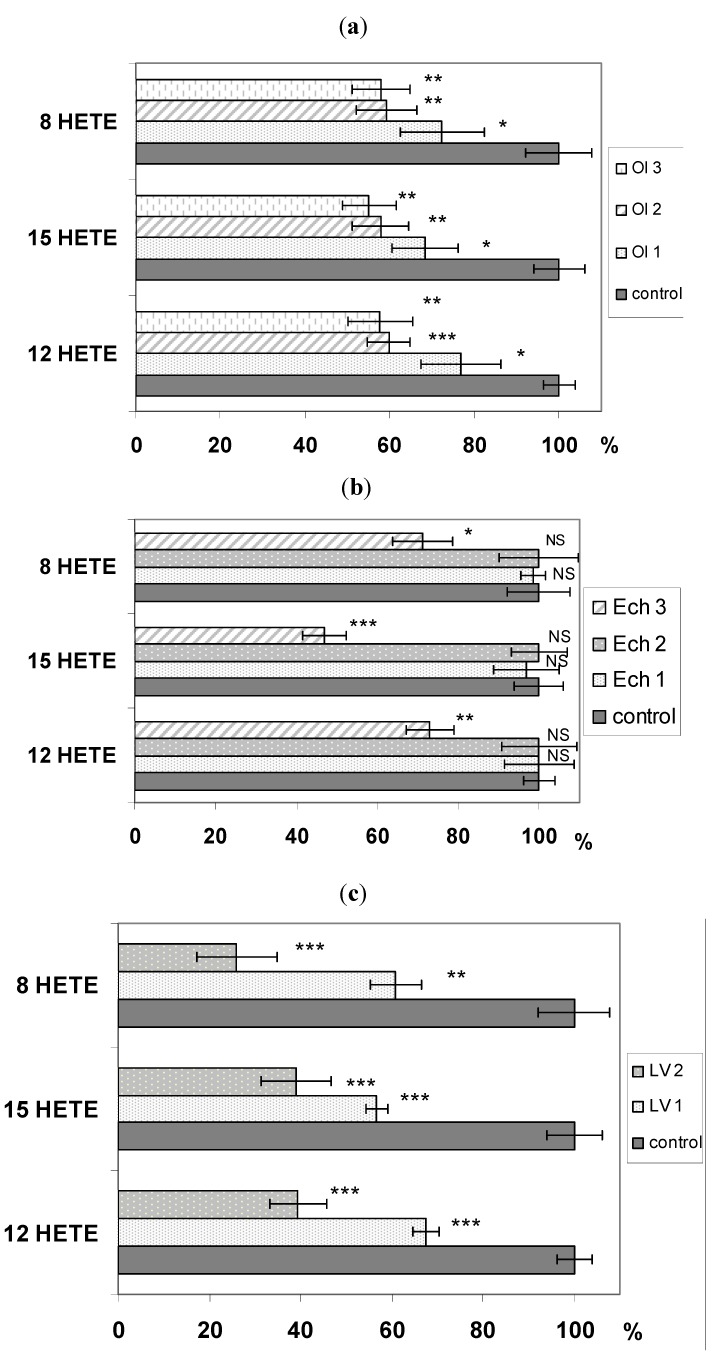
(**a**) Inhibitory activity of different concentrations of oleuropein on LOX; (**b**) Inhibitory activity of different concentrations of echinacoside on LOX; (**c**) Inhibitory activity of different concentrations of *Ligustrum vulgare infusion* on LOX. Each value is average ± SD of three separate experiments, * pt ˂ 0.05, ** pt ˂ 0.01, *** pt ˂ 0.001 (statistical significance for individual samples compared to those of the control).

**Table 2:** Concentrations of used inhibitors of LOX.

Sample	Oleuropein		Echinacoside		*Ligustrum vulgare* infusion	
	V (μL)	C (μg.mL^−1^)	V (μL)	C (μg.mL^−1^)	V (μL)	C (μg of lyophilizate.mL^−1^)
1	6.25	33.75	29.6	232.36	3.9	19.5
2	10.25	55.35	59.6	467.86	5.0	25.0
3	12.30	66.42	88.2	692.37	-	-

**Table 3:** SD values of measured antilipoxygenase activity for selected inhibitors.

Sample	Oleuropein			Echinacoside			*Ligustrum vulgare*		
	8-LOX 12-LOX 15-LOX			8-LOX 12-LOX 15-LOX			8-LOX 12-LOX 15-LOX		
control	7.8	3.8	6.1	7.8	3.8	6.1	7.8	3.8	6.1
1	9.9	9.5	7.9	2.9	8.6	8.1	5.5	2.9	2.3
2	7.1	5.1	6.7	9.8	9.2	6.9	8.8	6.2	7.8
3	6.9	7.6	6.4	7.4	5.9	5.5	-	-	-

*Ligustrum vulgare *L. is an abundant source of iridoids and phenolics [15]. HPLC-DAD profiles (Figure 2, right) show the complex polyphenol composition of *L*. *vulgare * leaves which include secoiridoids and tyrosol derivatives, and both hydroxycinnamates and flavonoid glycosides. Oleuropein, ligustaloside A and ligustaloside B, and ligstroside constitute the relevant secoiridoids class.

**Figure 2:**
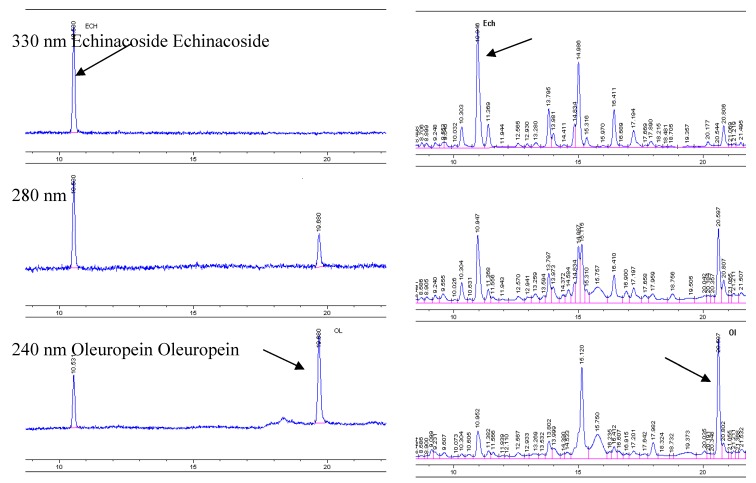
HPLC chromatograms of echinacoside and oleuropein standard solutions and a lyophilized water infusion of *Ligustrum vulgare *L. leaves*.* For HPLC conditions, see the Experimental section.

The flavonoid composition of *L*. *vulgare *leaves comprises quercetin 3-*O*-rutinoside, luteolin glucosides, namely luteolin 7-*O*-glucoside and luteolin 4'-*O*-glucoside and both apigenin 7-*O*-glucoside and apigenin 7-*O*-rutinoside. Hydroxycinnamates are represented by *p*-coumaric acid and echinacoside. Appreciable amounts of hydroxytyrosol and its glucoside were also detected in *L*. *vulgare *leaves. Antilipoxygenase activity can be potentiated with the activity of flavonoids (namely apigenin and luteolin derivatives) which exhibit remarkable activity against LOX [16]. Apigenin, luteolin, quercetin and their glycosides: Luteolin-7-rutinoside, quercetin-3-rutinoside, apigenin-7-rutinoside, luteolin-7-glucoside were previously isolated from *Ligustrum vulgare* [17], together with other plant polyphenols [9] such as hydroxytyrosol and its glucoside, the caffeic acid derivative acteoside and echinacoside or oleuropein. Our results suggest that an iridoid derivative, oleuropein, was an effective inhibitor of LOX*. *This is in good correlation with literature data. Oleuropein is one of the most active substances in virgin olive oil [18]. In intact rat peritoneal leukocytes stimulated with calcium ionophore, the principal phenolic compounds from the ‘polar fraction’: Oleuropein, tyrosol, hydroxytyrosol, and caffeic acid inhibited leukotriene LTB_(4)_ generation at the 5-lipoxygenase level with decreasing effectiveness hydroxytyrosol > oleuropein > caffeic acid > tyrosol.

Interaction of plant phenolics with mammalian lipoxygenases merits particular attention because these enzymes are potential targets for the health-preserving effects. Our current results and previous work, as well as data from the literature suggest that water infusion of *Ligustrum vulgare* leaves and its constituents are potent inhibitors of mammalian LOX.

## 3. Experimental Section

### 3.1. Plant Material

Leaves of *Ligustrum vulgare* L. were collected in the Arboretum Mlyňany, Institute of Dendrobiology, Slovak Academy of Sciences in September 2006 and dried at room temperature. Samples were identified by Dr. Tomaško (Arboretum Mlyňany) and a voucher specimen is deposited there.

### 3.2. Chromatography and Spectroscopy

#### 3.2.1. HPLC Determination of Oleuropein and Echinacoside in Water Infusion

Water infusion from leaves of *Ligustrum vulgare* L. was prepared by hot distilled water maceration (15 min, 1:10 plant-water weight ratio), and filtration after cooling. Cold infusion was lyophilized and from 100 g of an infusion 2.8582 g of lyophilizate sample were obtained. Analytical HPLC was performed with an Agilent Technologies HP 1050 Series with UV detector and autosampler (Agilent Technologies, Waldbronn, Germany). Compounds were separated on a 150 × 4.6 mm i.d., 5-μm particle, Zorbax Eclipse XDB C18 column (Agilent Technologies) using a gradient prepared from 0.1% aqueous H_3_PO_4_ (component A, Slavus s.r.o., Bratislava, Slovak Republic) and acetonitrile (component B, Sigma-Aldrich, Germany). The flow rate was 0.7 mL·min^−1^, the column temperature 28 °C and sample injection volume 20 μL. The gradient was: 0 min: 90% A + 10% B; 13 min: 78% A + 22% B; 23 min: 60% A + 40% B; 24 min: 90% A + 10% B. Echinacoside was purchased from PhytoLab (Vestenbergsgreuth, Germany) and oleuropein was isolated from the leaves of *Ligustrum vulgare* L. by the authors [9]. Figure 2 shows representative HPLC chromatograms of echinacoside and oleuropein standards and a sample of lyophilized water infusion of *Ligustrum vulgare* L. leaves. The UV spectra of two marker compounds were given by an UV detector, whereby the maximum absorption was set at 330 nm for echinacoside and 240 nm for oleuropein.

#### 3.2.2. Method Validation [19]

For two reference standards, good linear calibration curves were obtained as follows: Y = 21.183x − 10.086 (r^2^ = 0.9999) for echinacoside at concentrations of 1.42–100 μg·mL^−1^, Y = 22.559x − 9.9624 (r^2^ = 1) for oleuropein at concentrations of 1,27–100 μg·mL^−1^. The limits of detection (LOD) of standards were 1.09 μg·mL^−1^ for echinacoside and 1.08 μg·mL^−1^ for oleuropein. The limits of quantifications (LOQ) of standards were 1.42 μg·mL^−1^for echinacoside and 1.27 μg·mL^−1^for oleuropein. Good linear calibration curve was obtained for echinacoside at concentrations 1.69–72.29 μg·mL^−1^ and for oleuropein at concentrations 1.63–145.45 μg·mL^−1^ in the lyophilized infusion of *L. vulgare*. For the lyophilized infusion sample, LOD of echinacoside was 1.37 μg·mL^−1^ and for oleuropein 1.23 μg·mL^−1^, LOQ of echinacoside was 1.69 μg·mL^−1^ and for oleuropein 1.63 μg·mL^−1^. Thus, applied analytical procedure was suitable for the selected compounds quantification in *Ligustrum vulgare*.

#### 3.2.3. DPPH, ABTS and FRAP Tests

The antioxidative capacity of the lyophilizate was tested using three different methods, as described in the literature [20].

#### 3.2.4. HPLC Determination of Inhibiting Activity on Lipoxygenase

The cytosolic fraction from rat lung (Wistar rat, male 180 g) as a source of LOX was isolated according to procedure of Kulkarni *et al.* [21]. Briefly: Rat lung homogenate were centrifuged at 1,000 g for 5 min. The pellet that was obtained contained unbroken cells and debris was discarded. The resulting supernatant was centrifuged at 10,000 × g for 15 min to obtain the mitochondrial fraction. The post-mitochondrial supernatant was further centrifuged at 100,000 g for 60 min to obtain microsomes and cytosol. This fraction was further purified by ammonium sulphate precipitation (60%), chromatography on Sephadex G-150 and Phenyl-Sepharose CL-4B. Purified enzyme was used for LOX activity determination.The protein content in the enzyme preparation was estimated by the method of Bradford [22].

Arachidonic acid (AA, 99%, Sigma, St. Louis, MO, USA) was used as a substrate prepared in a solubilized state as described [23]. Standards of 8(*S*)-hydroxyeicosatetraenoic acid (8-HETE), 12(*S*)-HETE and 15(*S*)-HETE were purchased from Cayman Pharma s.r.o (Praha, Czech Republic).

Purified enzyme was analyzed for its ability to metabolize AA according to the literature [24]. Incubation (30 min, 100 rpm at ambient temperature) was conducted with the purified enzyme (25 μL) and 5% solution of AA (10 μL) Tris-HCl buffer (975 μL, pH 7.4) with or without (=control sample) presence of different amount of inhibitors (Table 2). Incubation was terminated by addition of sodium borohydride, which reduced the formed hydroperoxyacids to their more stable hydroxyderivatives. After acidification with concentrated HCl (100 μL) the sample was repeatedly centrifuged at 3000 × gfor 3 min with diethyl ether (1.5 mL). The supernatants were placed into a vial and evaporated to dryness in the nitrogen stream. After drying sample was reconstituted in the mobile phase used for HPLC analysis (acidified methanol:deionized water 85:15, 200 μL—See analytical HPLC below). The AA metabolites were identified comparing their retention times with those of authentic standards.

Analytical HPLC was performed with an Agilent Technologies HP 1,050 Series with UV detector and autosampler (Agilent Technologies). Compounds were separated on a 250 × 4.0 mm i.d., 5-μm particle, Nucleosil 120-5 C18 100A column (Watrex, Praha, Czech Republic) guarded with a Zorbax XDB C18, 12.5 × 4.6 mm pre-column (Agilent Technologies) using a gradient prepared from acidified methanol (0.5 mL of glacial acetic acid in 500 mL of methanol—component A) and deionized water (component B). The flow rate was 0.2 mL·min^−1^, the column temperature 28 °C and sample injection volume 50 μL. The gradient was: 0 min: 85% A + 15% B; 12 min: 100% A; 25 min: 85% A + 15% B; 27 min. UV detection at 210, 234 and 280 nm was used.

**Figure 3:**
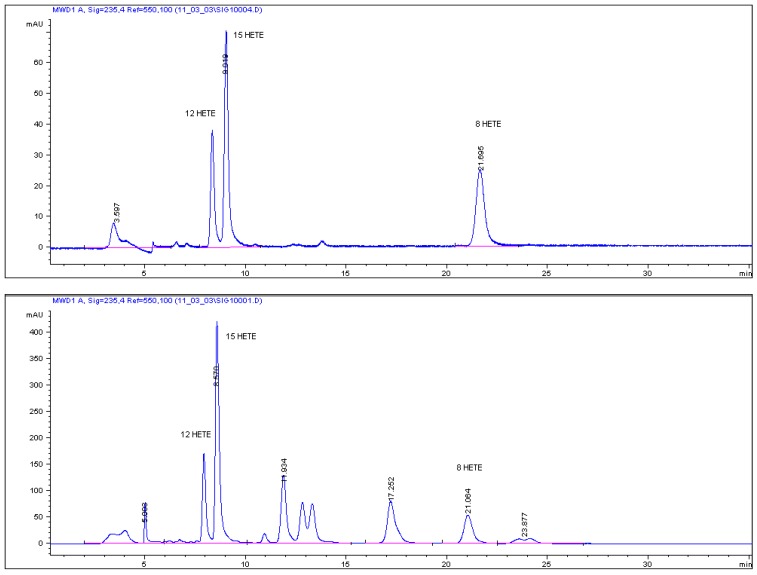
HPLC chromatogram of 8-,12- and 15-HETE standards (upper) and incubate of AA with purified enzyme (lower).

The eluate from the column between Rt = 19–22 min containing metabolites of AA was collected (data not shown). The collected fraction was evaporated under a nitrogen stream. After reconstitution in *n*-hexane the sample was analyzed by HPLC using the apparatus described above. Compounds were separated on a Zorbax Rx-SIL 150 × 2.1 mm i.d., 5-μm particle column (Agilent Technologies) using an isocratic elution with mobile phase prepared from *n*-hexane and acidified 2-propanol (0.5 mL of glacial acetic acid in 500 mL of 2-propanol in ratio 98:2. The flow rate was 0.15 mL·min^−1^, the column temperature was 28 °C and the sample injection volume 30 μL, with UV detection at 235 nm. The AA metabolites were identified by comparing their retention times with those of authentic standards and quantified by the peak area (Figure 3). For HPLC method validation ethanolic solutions of eicosanoid standard were evaporated to dryness under a nitrogen stream, redissolved in *n*-hexane at specified concentrations and used for creating the calibration curves.

For three reference standards, good linear calibration curves were obtained as follows: Y = 893.78x − 279.47 (r^2^ = 0.9925) for 8-HETE at concentrations of 0.139–2 μg·mL^−1^, Y = 604x − 177.91 (r^2^ = 0.998) for 12-HETE at concentrations of 0.144–4 μg·mL^−1^ and Y = 487.85x − 178.57 (r^2^ = 0.9979) for 15-HETE at concentrations 0.233–4.02 μg·mL^−1^. The LOD of standards were 0.134 μg·mL^−1^ for 8-HETE, 0.135 μg·mL^−1^ for 12-HETE and 0.195 μg·mL^−1^ for 15-HETE. The LOQ of standards were 0.139 μg·mL^−1^for 8-HETE, 0.144 μg·mL^−1^for 12-HETE and 0.233 μg·mL^−1^ for 15-HETE.

#### 3.2.5. Statistical Analysis

The statistical significances of all calculated values were determined by paired Student′s *t*-test (pt), The values represent the means ± standard deviation (SD).

## 4. Conclusions

In summary, this study and previous work reports the efficient inhibition of rat lung cytosolic fraction lipoxygenase by a water infusion of *Ligustrum vulgare* leaves and two its constituents*.* These results indicate that the tested samples may contribute to the activity of *L*. *vulgare *when used as an anti-inflammatory agent in traditional medicine. However, the prospective therapeutic value and the real clinical efficacy of this plant should be proved by further *in vitro *and *in vivo *experiments.
